# The Parotoid Gland Secretion from Peruvian Toad *Rhinella horribilis* (Wiegmann, 1833): Chemical Composition and Effect on the Proliferation and Migration of Lung Cancer Cells

**DOI:** 10.3390/toxins12090608

**Published:** 2020-09-22

**Authors:** Guillermo Schmeda-Hirschmann, Jean Paulo de Andrade, Marilú Roxana Soto-Vasquez, Paul Alan Arkin Alvarado-García, Charlotte Palominos, Sebastián Fuentes-Retamal, Mathias Mellado, Pablo Correa, Félix A. Urra

**Affiliations:** 1 Laboratorio de Química de Productos Naturales, Instituto de Química de Recursos Naturales, Universidad de Talca, Campus Lircay, Talca 3460000, Chile; jean.deandrade@utalca.cl; 2 Núcleo Científico Multidisciplinario, Dirección de Investigación, Universidad de Talca, Campus Lircay, Talca 3460000, Chile; 3 Laboratorio de Farmacognosia, Facultad de Farmacia y Bioquímica, Universidad Nacional de Trujillo, Trujillo 13011, Peru; msoto@unitru.edu.pe; 4 Facultad de Medicina, Universidad Privada Antenor Orrego, Trujillo 13001, Peru; palvaradog1@upao.edu.pe; 5 Laboratorio de Plasticidad Metabólica y Bioenergética, Programa de Farmacología Clínica y Molecular, Instituto de Ciencias Biomédicas, Facultad de Medicina, Universidad de Chile, Santiago 8380453, Chile; palominos.ch@gmail.com (C.P.); sebastianfuentesr@gmail.com (S.F.-R.); mathignaciomellado@gmail.com (M.M.); pablo.correa.r@gmail.com (P.C.); 6 Network for Snake Venom Research and Drug Discovery, Santiago 7800003, Chile

**Keywords:** *Rhinella horribilis*, toad toxins, Peru, cell migration, proliferation, etoposide, bufadienolides

## Abstract

Since *Rhinella* sp. toads produce bioactive substances, some species have been used in traditional medicine and magical practices by ancient cultures in Peru. During several decades, the *Rhinella horribilis* toad was confused with the invasive toad *Rhinella marina*, a species documented with extensive toxinological studies. In contrast, the chemical composition and biological effects of the parotoid gland secretions (PGS) remain still unknown for *R. horribilis*. In this work, we determine for the first time 55 compounds from the PGS of *R. horribilis*, which were identified using HPLC-MS/MS. The crude extract inhibited the proliferation of A549 cancer cells with IC_50_ values of 0.031 ± 0.007 and 0.015 ± 0.001 µg/mL at 24 and 48 h of exposure, respectively. Moreover, it inhibited the clonogenic capacity, increased ROS levels, and prevented the etoposide-induced apoptosis, suggesting that the effect of *R. horribilis* poison secretion was by cell cycle blocking before of G2/M-phase checkpoint. Fraction B was the most active and strongly inhibited cancer cell migration. Our results indicate that the PGS of *R. horribilis* are composed of alkaloids, bufadienolides, and argininyl diacids derivatives, inhibiting the proliferation and migration of A549 cells.

## 1. Introduction

**Figure 1:**
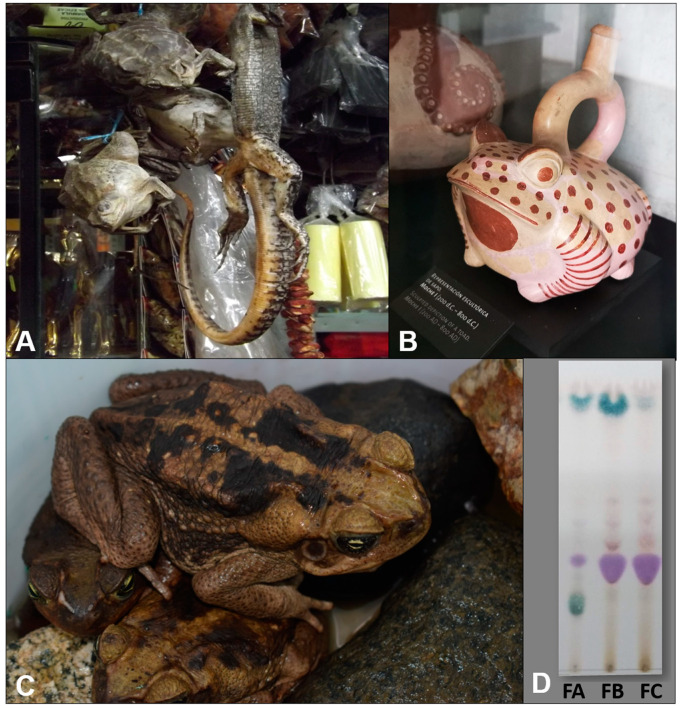
Representations of *Rhinella* species in traditional medicine and magical practices in Peru: (**A**) frogs and lizards on sale at the “Mercado de Brujos” (Witch’s market), Chichlayo, Peru; (**B**) Moche ceramic representing a frog; (**C**) adult specimen of *Rhinella horribilis* (Wiegmann, 1833), Peru; (**D**) thin layer chromatographic analysis (TLC) of the Sephadex LH-20 permeation showing the fraction pools A (FA; 27–30), B (FB; 31–38), and C (FC; 39–57) of PGS from *R. horribilis*. Adsorbent: silica gel; solvent system: EtOAc:isopropanol:NH_4_OH (9:7:4). The plate was sprayed with a freshly prepared *p*-anisaldehyde solution reagent followed by heating up to 105 °C for maximum visualization of spots.

Toads are widely distributed in the different biotas of Peru with a higher number of species in the Amazonian and eastern Andes [1,2] and are less frequent in the dry coastal ecosystems. Toads were known by the Native Americans both for their role in controlling insects as well as by their toxic defense substances. These toad toxins are secreted from parotoid glands that in contact with oral mucosa or eyes can produce intoxication or death [3,4,5]. Ceramic representations of *Rhinella* toad species by Peruvian pre-Inca Chavin and Moche cultures [6,7] and current local magic traditions in northern Peru that involved these toads (Figure 1A,B) suggest the presence of bioactive compounds. Currently, no studies are reporting the chemical composition and biological effects of the parotid glands secretions (PGS) of *Rhinella* sp. toads of the northern coast of Peru. The PGS of South American toads include alkaloids, argininyl diacids, bufadienolides, and argininyl diacid derivatives from bufadienolides. The biological effects from these secretions include cardiotonic, cytotoxic, and antiproliferative activities, sometimes with a remarkable and selective effect on several cancer cell lines [8,9,10].

Non-small cell lung cancer (NSCLC) is the leading cause of cancer-related deaths globally [11]. The NSCLC tumors have high molecular heterogeneity, exhibiting gene mutations in B-Raf proto-oncogene, serine/threonine kinase *(BRAF)*, *KRAS* proto-oncogene, and activating mutations of epidermal growth factor receptor (EGFR) [12]. Although recent advances in the development of new therapeutic strategies are focused on the reduction of proliferation and tumor growth [13,14]; reduced efforts have been put into identifying anti-invasive and anti-migratory drugs [15]. Promising uses of microtubule-targeting agents (MTA) have been proposed for NSCLC treatment in advanced stages [13]. Beyond the known effects of MTA on cell cycle progression, the blocking the microtubules stabilization by these anti-cancer drugs produces disruption of cell protrusions and cytoskeleton, triggering inhibition of tumor-induced angiogenesis and cancer cell migration/invasion [16].

Previously, we and others have described that the PGS of *Rhinella* species produces inhibition of proliferation by cell cycle arrest and induction of apoptosis [9,17,18], being the bufadienolides a promising source of anti-cancer chemical scaffolds [19]. The South American toad defense secretions consist of a complex mixture of argininyl diacids, bufadienolides argininyl diacids, bufadienolides, indolealkylamines, and peptides. The components less investigated so far are the peptides. Bioactive peptides also are considered a potential class of anticancer natural products, which inhibit cancer proliferation [20,21], angiogenesis [22], and induce selective apoptosis [23].

Little is known on the PGS composition and bioactivity of toad defensive compounds for the western South American Andes. No information is available on the secretion constituents of the recently known species of the northern Peru *Rhinella horribilis* (Figure 1C), which was extensively confused with *Rhinella marina* [24], a highly invasive species with well-known toxicological effects and bioactivities [8,18,25,26].

In this work, we report for the first time the chemical composition of the PGS from the Peruvian toad *R. horribilis* and describe the effect of the whole secretion and their main fractions on human lung cancer cells.

## 2. Results and Discussion

### 2.1. Fractionation of Parotoid Gland Secretions of R. horribilis

The crude secretions were fractionated into a group of constituents according to molecular size by permeation on Sephadex LH-20. The sample (660 mg) was loaded into a Sephadex LH-20 column (column length: 28.5 cm; internal diameter: 3.5 cm; filled with Sephadex^®^ LH-20, Merck KGaA, Darmstadt, Germany). The eluent was methanol:chloroform (1:1). The void volume was 300 mL and 95 fractions of 3 mL each were collected. After TLC analysis (solvent system: ethanol:isopropanol:ammonium hydroxide 9:7:4 *v*/*v*/*v*), fractions with similar patterns were pooled as follows: fraction 11–26 (182.6 mg), fraction 27–30 (96.6 mg), fraction 31–38 (116.8 mg), fraction 39–57 (77.3 mg), fraction 58–69 (12 mg), and fraction 70–95 (12 mg) (Figure 1D). The pooled fractions containing most of the constituents were further analyzed by ^1^H NMR to obtain structural information on the group of compounds and for further examination by HPLC-MS/MS.

### 2.2. ^1^H NMR Analysis

The ^1^HNMR spectrum of the fractions 27–30 (Pool A) showed the characteristic signals for the H-21, H-22, and H-23 protons of the bufadienolide *α*-pyrone ring at δ 7.37–7.48, 7.86–8.00, and 6.25–6.32 ppm, respectively. Minor compounds of the pool presented a singlet at δ 9.67, 9.58, and 9.48 ppm, corresponding to an aldehyde function. The less abundant bufadienolides in the complex mixture displayed a singlet or broad singlet at δ 10.10, 10.08, 10.07, and 10.03 ppm, compatible with a second group of oxidized derivatives with an aldehyde group. According to Kamano et al. [27], the aldehyde signal resonates at δ 10.06 ppm for hellebrigenin and δ 9.49 ppm for resibufagin. The ^1^HNMR spectrum of our sample supports the presence of a mixture of compounds belonging mainly to the bufalin and marinobufagin series, with the aldehydes and further oxidized bufadienolides as minor constituents. The main bufadienolides in the fraction are marinobufagin and bufalin argininyl diacids. The HPLC-MS/MS analysis (Section 2.3) confirmed the occurrence of several hellebrigenin and resibufagin argininyl diacids, supporting the NMR assignments. The Pool B, including fractions 31–38, shows a mixture of argininyl diacids, free bufadienolides, and minor amounts of bufadienolides argininyl diacids. The main groups of the components were resibufagin, telocinobufagin, marinobufagin, bufalin, and resibufogenin derivatives. The identity was established by HPLC-MS/MS analysis (see Section 2.3). The ^1^HNMR analysis of the fraction 39–57 (Pool C) showed a main compound with H signals at δ 7.32 d (8.4) (1H), 7.12 s (1H), 6.86 d (8.4) (1H), 4.02 br t (5.6) (2H), 3.71 s (6H) and 3.31 ppm (br t, 5.6), in agreement with dehydrobufotenine (DHB) (compound A2). Additional signals support the presence of a mixture of argininyl diacids and minor amounts of bufadienolides. HPLC-MS/MS analysis of the fraction confirmed the occurrence of DHB as the main constituent and the presence of adipyl-, pimeloyl- and suberoylarginine. The ^1^HNMR analysis of the pool of fractions 58–69 and 70–95 showed the characteristic signals for a mixture of argininyl diacids and was not further investigated.

### 2.3. HPLC-DAD-Q-TOF-MS/MS Analysis

The crude PGS and the main fraction pools A, B, and C were analyzed by high performance liquid chromatography (HPLC) coupled to a diode-array detector (DAD) and to a quadrupole time-of-flight mass spectrometry/mass spectrometry detector (Q-TOF-MS/MS) to identify the constituents of the complex mixtures (Table 1). Detected and calculated [M+H]^+^ ions were compared and the error was calculated for the tentative identification of the different compounds. Data analyses followed previous work on toad poison secretions [9,10,18,28,29,30,31].

#### 2.3.1. Compounds Identified: Argininyl Diacids

**Figure 2:**
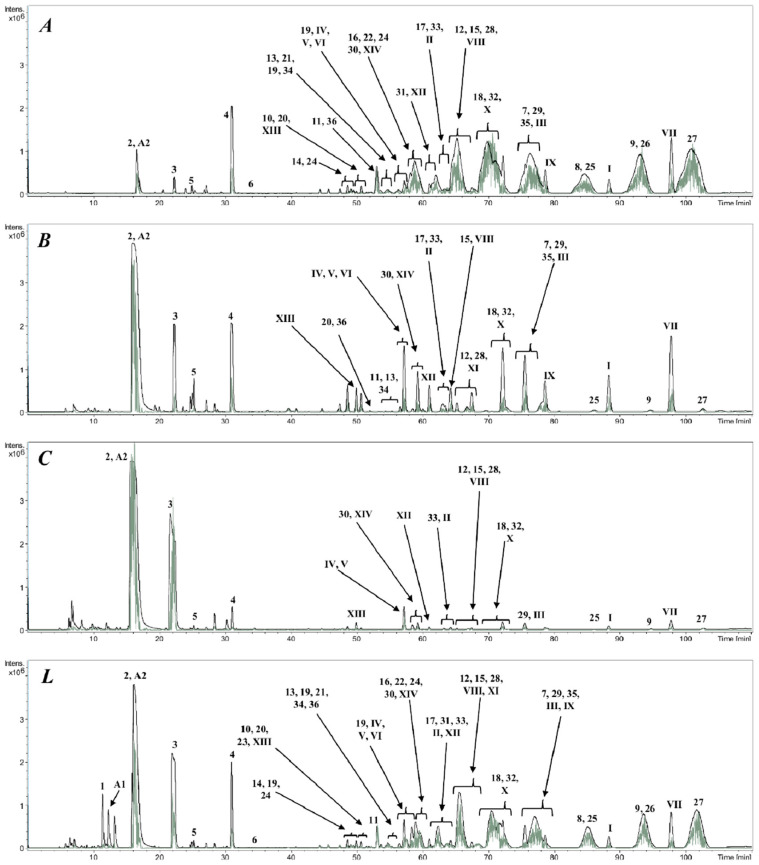
HPLC-MS/MS chromatogram of the fractions 27–30 (A), 31–38 (B), 39–57 (C), and the lyophilized crude extract (L) of the parotoid gland secretions from Peruvian *Rhinella horribilis*. Total ion current (TIC) (black line) and MS^2^ (gray line). Detection in the positive ion mode.

**Figure 3:**
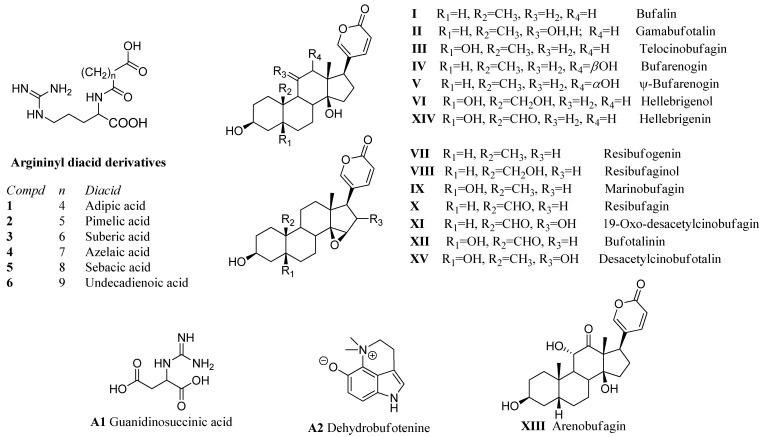
Structure of the compounds A1, A2, genines (bufadienolides) I–XV, and argininyl diacids **1**–**6** tentatively identified in PGS of *R. horribilis*.

In the HPLC-MS/MS analysis (Table 1 and Figure 2), six compounds eluting in the first third of the chromatographic run showed the loss of a diacid from the positively charged ion and arginine at *m/z* 175 as the common amino acid in the structural series. The difference in the compounds **1**–**6** (Figure 3) was the number of CH_2_ units in the diacid moiety. The compounds were identified as adipoyl-, pimeloyl-, suberoyl-, azelayl-, sebacyl- and undecadienoyl arginine, respectively. Argininyl diacids were described in the poison secretions of other South American *Rhinella* species, including *R. marina* [18], *R. schneideri* [9,10], *R. ornata* and *R. scitula* [9]. For all argininyl diacids and *N*-diacid argininyl bufadienolides, extracted ion chromatograms at *m/z* 303, 317, 331, 345, 359 and 373 allowed to confirm the occurrence of adipyl-, pimeloyl-, suberoyl-, azeloyl-, sebacyl- and undecadienoyl arginine esters, respectively.

#### 2.3.2. Alkaloids and Guanidine Derivatives

The alkaloid guanidinosuccinic acid (A1) and dehydrobufotenine (A2) were detected in the PGS (Figure 3). The fragmentation pattern and errors comparing the detected and expected mass fully support the identification. Dehydrobufotenin was reported in the PGS of *R. marina* and *R. schneideri* [9,18].

#### 2.3.3. Bufadienolides

The bufadienolides bufalin (I), gamabufotalin (II), telocinobufagin (III), bufarenogin/ψ-bufarenogin (IV, V), hellebrigenol (VI), resibufogenin (VII), resibufaginol (VIII), marinobufagin (IX), resibufagin (X), 19-oxo-desacetylcinobufagin (XI), bufotalinin (XII), arenobufagin (XIII), and hellebrigenin (XIV) were identified by comparing the molecular formula and fragmentation patterns with literature. According to Ye and Guo [29], resibufaginol (VIII) elutes before marinobufagin (IX) and, the elution sequence agrees with our assignment (Figure 3).

#### 2.3.4. Argininyl Diacids of Bufadienolides

**Figure 4:**
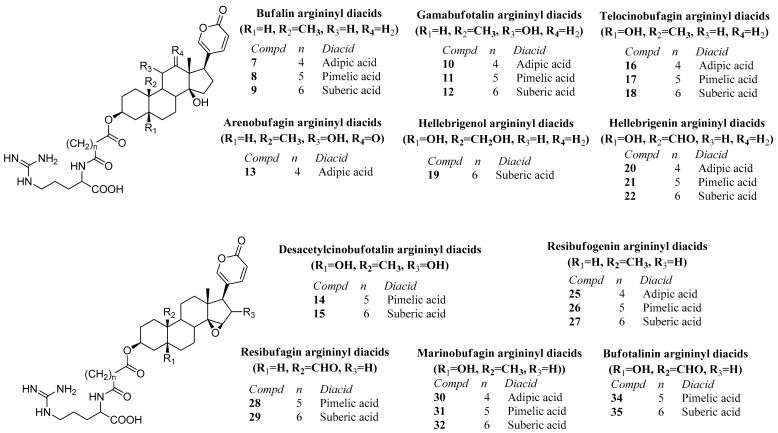
Structure of the compounds **7**–**22**, **25**–**32**, and **34**–**35** tentatively identified in the parotoid gland secretion of *R. horribilis*.

In the toad secretions, bufadienolides occur as free and as argininyl diacid derivatives. The identity of the bufadienolide moiety followed from the analysis of the neutral loss of water from the [M+H]^+^ ion and subsequent fragmentation leading to the arginine on at *m*/*z* 175. The neutral loss of 368 amu from the [M+H]^+^ ion was used to identify the homologous series of bufalin derivatives. Compounds **7**, **8**, and **9** were identified as 3-(*N*-adipoylargininyl)-, 3-(*N*-pimeloylargininyl)- and 3-(*N*-suberoylargininyl) bufalin, respectively, in agreement with the literature [9]. The telocinobufagin derivatives, with an additional hydroxyl function at C-5, showed a constant neutral loss of 384 amu, as can be observed for the mass spectra of compounds **16**–**18**. While the identity of the argininyl diacid moiety can be unambiguously assigned, telocinobufagin and its isomer gamabufotalin, with a hydroxyl function at C-11 instead of C-5, show the same molecular formula and fragments. In the *R. horribilis* poison secretion, two compounds with the molecular formula C_36_H_54_N_4_O_9_ were detected at R_t_ 49.8–50.1 and 57.9–58.2 min; two products with the molecular formula C_37_H_56_N_4_O_9_ at R_t_ 53.3–54.1 and 62.4–64.7 min; and two compounds presenting the molecular formula C_38_H_58_N_4_O_9_ at R_t_ 66.4–67.2 and 68.6–72.0 min, and were assigned to the adipoyl- (compound **10** and **16**), pimeloyl- (**11** and **17**) and suberoyl- (compounds **12** and **18)** esters of gamabufotalin and telocinobufagin, respectively. According to Cao et al. [28] for argininyl diacids from gamabufotalin and telocinobufagin, the homologous series have higher Rt for the telocinobufagin than for the gamabufotalin argininyl diesters. Therefore, in the pairs with the same molecular formula and fragments, the compound with lower Rt was tentatively identified as the gamabufotalin argininyl ester. The same observation regarding Rt values is found for the genines by Ye and Guo [29] and Ren et al. [33]. Three compounds with molecular formula C_38_H_58_N_4_O_10_ eluted at R_t_ 49.1–49.2, 54.2–54.6 and 57.0–57.8 min and fragmented to the base peak at *m*/*z* 331, in agreement with 3-(*N*-suberoyl argininyl) hellebrigenol (compound **19**). The three compounds differ in the intensity of the ions after fragmentation and were assigned as suberoyl argininyl hellebrigenol isomers 1, 2, and 3, respectively (Figure 4).

Compounds **20**–**22** are isomers of the compounds **13**–**15**, differing in the placement of the hydroxy function (C-5 for **20**–**22**, C-11 for **13,** and C-5/C-16 for **14**–**15**). The compounds **14** and **15** present an epoxy function while there is a carbonyl ketone at C-12 in **13** and the aldehyde from hellebrigenin in compounds **20**–**22**. The compounds **20**–**22** were assigned as adipoyl-, pimeloyl- and suberoylargininyl hellebrigenin based on the neutral loss of 398 amu, leading to the peak of the argininyl diacids. 3-(*N*-suberoylargininyl) hellebrigenin (hellebritoxin) was reported from the PGS of *R. marina* from the Peruvian Amazon [18] and Paraguayan *Rhinella* species [9].

The occurrence of hellebrigenin and resibufagin argininyl diacids in our samples was suggested by the ^1^HNMR analysis of the PGS and fractions. The isomer pairs with the molecular formula C_36_H_52_N_4_O_10_, C_37_H_54_N_4_O_10,_ and C_38_H_56_N_4_O_10_, were assigned as 3-(*N*-adipoyl argininyl) arenobufagin **13** and 3-(*N*-adipoyl argininyl) hellebrigenin **20**; 3-(*N*-pimeloyl argininyl) desacetylcinobufotalin **14** and 3-(*N*-pimeloyl argininyl) hellebrigenin **21**; 3-(*N*-suberoyl argininyl) desacetylcinobufotalin **15** and 3-(*N*-suberoyl argininyl) hellebrigenin **22**, respectively. The assignment of the arenobufagin and hellebrigenin argininyl diacids in our samples of PGS follows the elution sequence previously reported for the genines and argininyl esters of both compounds, with hellebrigenin isomers eluting before the corresponding arenobufagin derivatives [33]. Ren et al. [33] reported that arenobufagin elutes before hellebrigenin in their PGS samples. Furthermore, the loss of 29 amu in the hellebrigenin derivatives was observed for compounds **20**–**22** in agreement with Ye and Guo [29] (Figure 4).

**Figure 5:**
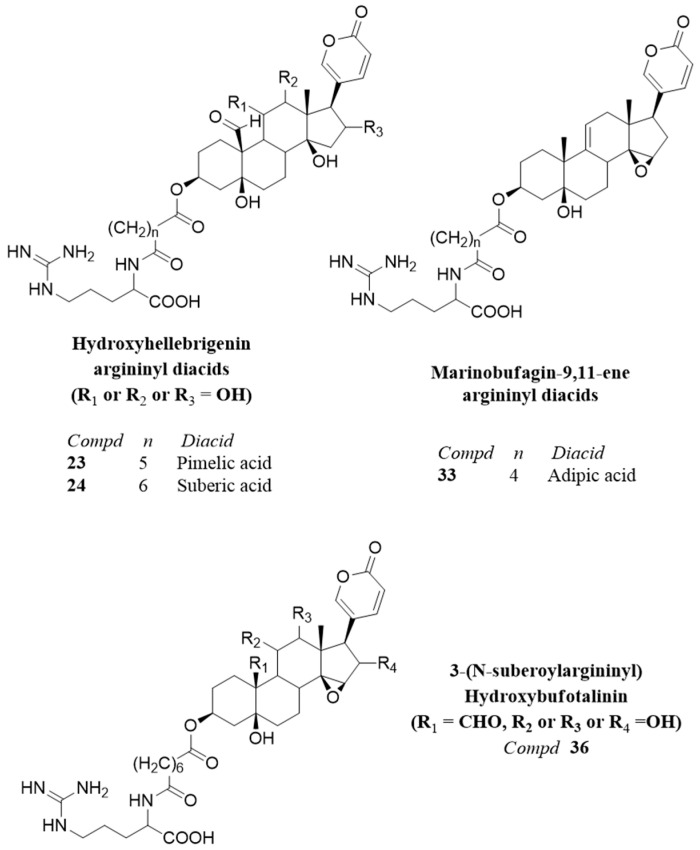
Structure of the compounds **23**–**24**, **33,** and **36** tentatively identified in the parotoid gland secretion of *R. horribilis*.

The compound **23** showed the neutral loss of 414 amu and a base peak in agreement with pimeloylarginine and was assigned as 3-(*N*-pimeloyl argininyl) hydroxyhellebrigenin. Two compounds with the same molecular formula C_38_H_56_N_4_O_11_ and R_t_ 48.8–49.3 and 58.1–58.4 min occur in the PGS. The mass spectrum of the compound, described here as compound **24**, showed the neutral loss of 414 amu, leading to the suberoylargininyl ion at *m*/*z* 331. The compound was assigned as 3-(*N*-suberoyl argininyl) hydroxyhellebrigenin. The placement of the additional hydroxyl function in compound **24** remains to be established, being C-12 or C-16 the most probable options. The isomers were tentatively identified as 3-(*N*-suberoyl argininyl) hydroxyhellebrigenin **24** isomer 1 and 2, respectively. The resibufogenin derivatives (compounds **25**, **26** and **27**) were assigned based on the loss of 366 amu from the [M+H]^+^ ion, leading to the argininyl diacids and were identified as adipoyl-, pimeloy- and suberoylresibufogenin, respectively. The compounds were previously reported from the Amazonian *R. marina* [18] and Paraguayan *Rhinella* species [9] (Figure 4 and Figure 5).

The compounds **28**, **29**, **34,** and **35** were tentatively assigned by the neutral loss of the bufadienolide from the [M+H]^+^ ions, leading to the argininyl esters. The compounds **28** and **29** with a neutral loss of 380 amu are compatible with the aldehyde derivatives of resibufogenin (resibufagin) and were assigned as pimeloylargininyl resibufagin **28** and suberoylargininyl resibufagin **29**, respectively. The compounds **34** and **35** showed the neutral loss of 396 amu in agreement with aldehyde derivatives of marinobufagin (bufotalinin), being assigned as pimeloylargininyl bufotalinin **34** and suberoylargininyl bufotalinin **35,** respectively. The compounds **30**, **31,** and **32** showed the neutral loss of 382 amu, with base peak for adipoyl-, pimeloyl-, and suberoylarginine and were identified as marinobufagin argininyl diacids, in full agreement with spectrometric data reported for South American *Rhinella* species [10,18]. Marinobufagin is the 5-hydroxy derivative of resibufogenin. The compound **33**, with the molecular formula C_36_H_50_N_4_O_9_ shows one additional unsaturation than compound **30**, it presents the neutral loss of 380 amu and the base peak for adipoyl arginine. It was tentatively assigned as 3-(*N*-adipoyl argininyl) marinobufagin-9,11-ene **33**. Two compounds with the molecular formula C_38_H_54_N_4_O_11_ eluted at R_t_ 52.1–52.4 and 54.7–54.9 min, respectively, and showed the neutral loss of 412 amu, suggesting the occurrence of hydroxybufotalinin derivatives differing in the placement of the hydroxyl group. For hydroxybufotalinin, the most common hydroxylation places are either at C-11, C-12, or C-16. However, we are not able to fully assign the most probable isomers. The compound **36** was tentatively identified as 3-(*N*-suberoyl argininyl) hydroxybufotalinin **36** isomers 1 and 2, respectively (Figure 4 and Figure 5).

In this study, we identified a high number of constituents (55 compounds) from PGS of *R. horribilis* compared to the 29 compounds previously reported from three Peruvian Amazon populations of *R. marina* [18]. Although this difference might be explained by the different environments, access to food or the sensibility of the instrument used, we are not excluding the hypothesis of unique chemical composition patterns for both species. This is especially relevant if is considered that both *R. marina* and, previously confused *R. horribilis*, have geographical, genetic, and morphological differences [24,34]. The main compounds in the PGS are marinobufagin and bufalin derivatives, with aldehydes as minor constituents of the mixture. Oxidation of the methyl group at C-10 led to the primary alcohols and then to the corresponding aldehydes, giving origin to the bufotalinin derivatives starting from marinobufagin, resibufagin derivatives from resibufogenin, and hellebrigenin from telocinobufagin, respectively. Further oxidation of the steroid moiety led to alcohol and ketone functions mainly at C-11, C-12, or C-16. The unambiguous identification of the minor constituents requires isolation by preparative HPLC and extensive spectroscopic and spectrometric studies to identify the exact position and stereochemistry of the functional groups. Some differences with the Peruvian *R. marina* are the occurrence of arenobufagin derivatives and a higher number of oxidized compounds in *R. horribilis* compared with the Amazonian species. Interestingly, most of the compounds reported in the *R. horribilis* PGS have been previously described from South American *Rhinella* species [9,10,18] and/or from the crude drug Venenum Bufonis or Ch’an Su from China and Eastern Asia [27,30,31], which exhibit anti-cancer effects in several cancer cell lines [35,36,37,38,39,40]. Based on this, we propose to evaluate the anti-cancer effect of crude PGS of *R. horribilis* and their fractions A, B, and C.

### 2.4. Effect of R. horribilis PGS on Proliferation, Clonogenic Capacity and Reactive Oxygen Species (ROS) Levels of Lung Cancer Cells

**Figure 6:**
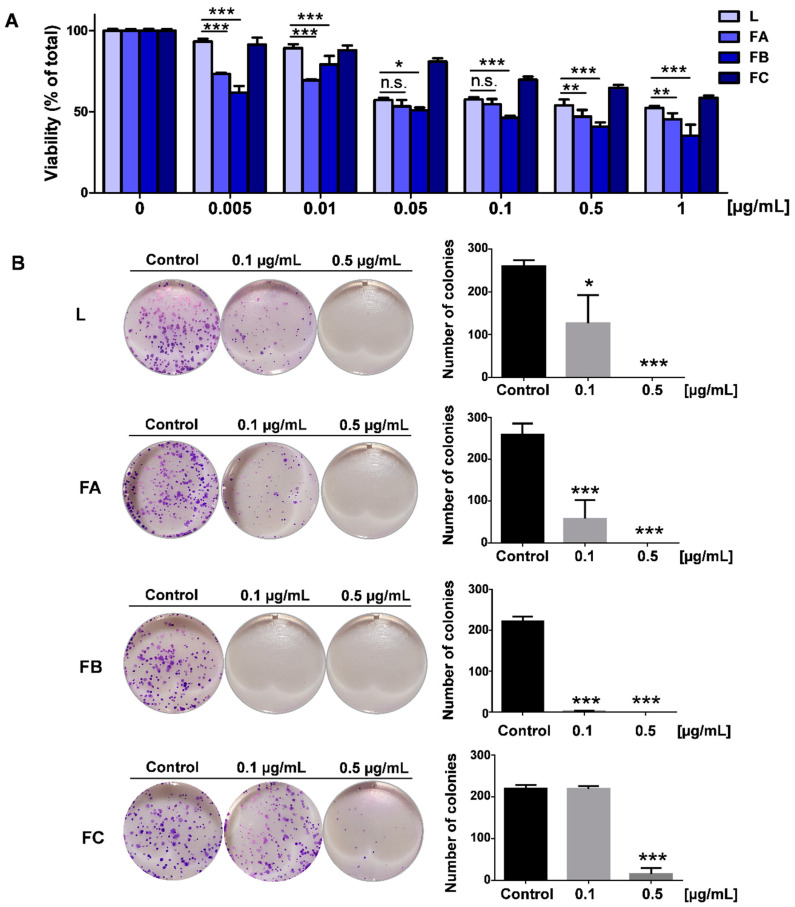
Effect of fractions A, B, C (FA, FB, FC) and crude extract (L) of parotoid gland secretion (PGS) of *R. horribilis* on viability and clonogenic potential of lung cancer A549 cells. (**A**) Effect of fractions and crude extract on viability at 24 h of exposure. (**B**) Cells were exposed 24 h to DMSO (control), crude extract (L), and fractions (A, B, and C); then, the culture medium was removed and replaced by fresh medium. The number of colonies was determined at 7 days, using crystal violet staining. Data shown are the mean ± SEM of three independent experiments. * *p* < 0.05, ** *p* < 0.01, *** *p* < 0.001, vs. control (DMSO); n.s., not significant.

To evaluate the anti-proliferative activity of crude PGS (L) of *R. horribilis* and their fractions A, B, and C, lung cancer A549 cells were treated with increasing concentrations by 24 and 48 h and the IC_50_ values were calculated. As Table 2 shows, the crude PGS had an IC_50_ value of 0.031 ± 0.007 µg/mL at 24 h, and fractions A, B, and C were close to 3.08, 4.4, and 1.26 folds more active than crude PGS, respectively and this was a concentration-dependent effect (Figure 6A). At 48 h, fraction C exhibited a strong increase in its anti-proliferative activity.

**Figure 7:**
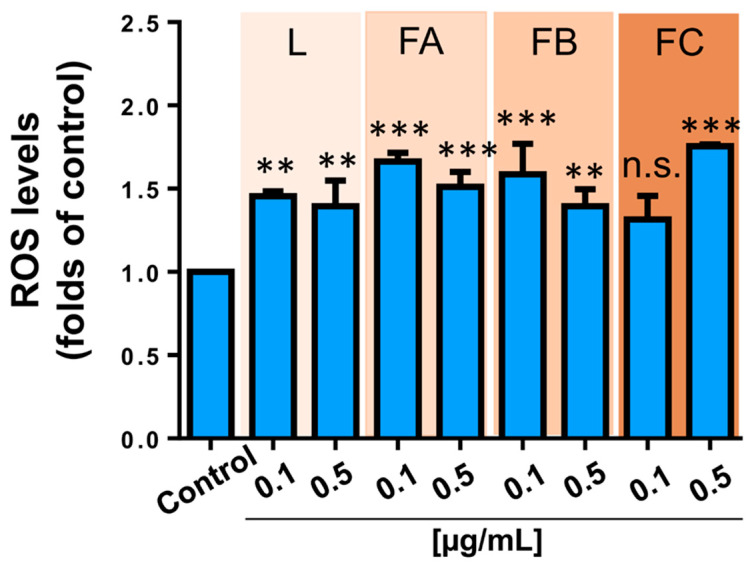
Effect of fractions and crude extract of parotoid gland secretions of *R. horribilis* on ROS production in A549 cancer cells. Cells were exposed 24 h to DMSO (control), crude extract (L), and fractions (A, B, and C), and changes in the ROS levels were determined using dihydroethidium (DHE) probe by flow cytometry. Data shown are the mean ± SEM of three independent experiments. ** *p* < 0.01, *** *p* < 0.001, vs. control (DMSO). n.s. not significant.

The colony formation is the ability of a single cancer cell to proliferate indefinitely, growing into a colony [41]. Since that the concentrations 0.1 and 0.5 µg/mL shown significant differences in viability (Figure 6A), we evaluate if the crude PGS and theirs fraction A, B, and C irreversibly affect the clonogenic potential of A549 cancer cells. These cells were treated for 24 h with 0.1 and 0.5 µg/mL of crude PGS and their fractions and then, the culture medium was washed and replaced by a fresh and PGS-free culture medium for seven days. As Figure 6B shows, fraction B evoked a strong and irreversible inhibition of the clonogenic potential and fraction C was only active at 0.5 µg/mL. Moreover, we evaluate the effect of the *R. horribilis* poison secretion and their fractions on reactive oxygen species (ROS) levels in A549 cancer cells. As Figure 7 shows, all the conditions produced a significant increase in the intracellular ROS levels. Taken together, these results suggest that the PGS of *R. horribilis* increases ROS production and inhibits the proliferation and clonogenic capacity of lung cancer cells, being the fraction B the most active.

### 2.5. Effect of R. horribilis PGS and Their Fractions on Etoposide-Induced A549 Cell Death

Etoposide (Eto) is a known topoisomerase II inhibitor used in the treatment of several solid cancer, inducing DNA damage, growth arrest, and apoptosis in a concentration-dependent manner [42]. In several cancer cell lines, Eto produces cell cycle arrest in G2-phase [43] through p53 stabilization [44], promoting apoptosis [45]. On the other hand, we have described that the poison secretions of *Rhinella schneideri*, *R. ornata*, and *R. marina* produce G1- or S-phase arrest [9,18], avoiding the cell cycle progression to the G2-phase checkpoint. Therefore, to determine the action of poison secretions of *R. horribilis* on phases of the cell cycle, we evaluate its effect (at 0.5 µg/mL) on G2/M arrest-induced cell death by Eto. We hypothesized that whether the *R. horribilis* poison secretion acts on the cell cycle in an earlier phase different to G2/M phase, it will produce a systematic decrease of Eto-induced cell death.

**Figure 8:**
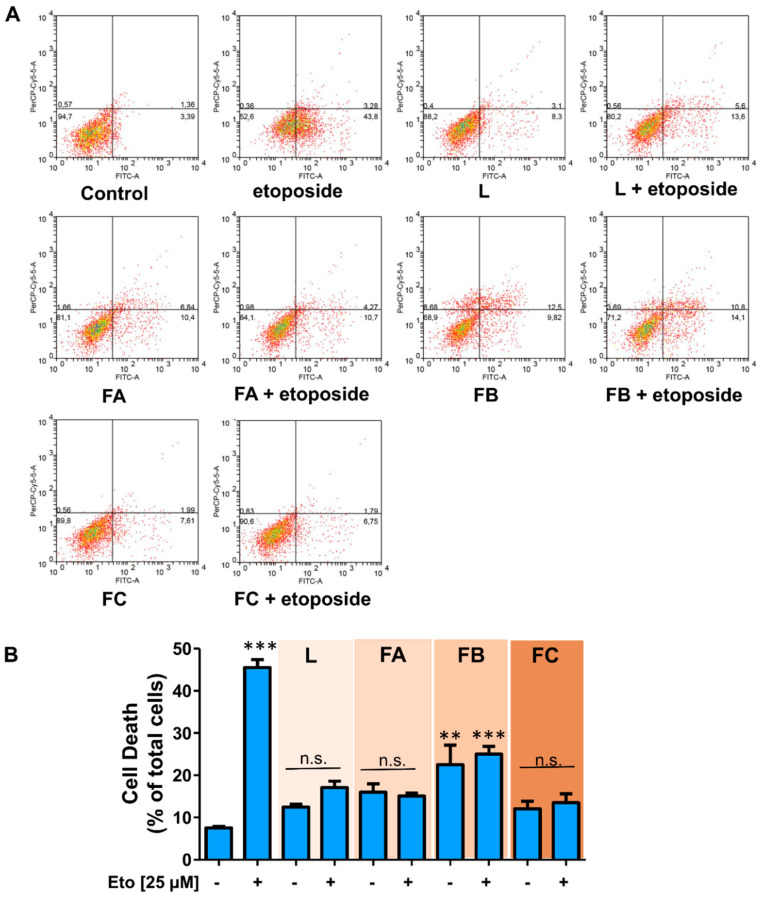
Effect of fractions A, B, C, and crude extract of parotoid gland secretions of *R. horribilis* on etoposide-induced cell death in A549 cancer cells. Cells were exposed 48 h to DMSO (control), crude extract (L), and fractions (FA, FB, and FC) at 0.5 µg/mL in the presence and absence of 25 µM etoposide (Eto), and viability were determined using Annexin V/P.I. assay by flow cytometry. (**A**) Representative dot-plots; (**B**) quantification. Data shown are the mean ± SEM of three independent experiments. ** *p* < 0.01, *** *p* < 0.001, vs. control (DMSO). n.s. not significant.

At 48 h of treatment, 25 µM Eto produced 45.50 ± 1.38% of positive annexin V subpopulation (*p* < 0.001 vs. control), suggesting apoptotic cell death as described [46,47]. As Figure 8 shows, only fraction B induced 22.51 ± 3.32% of apoptotic cancer cells and crude PGS and other fractions lacked cytotoxic effect. As it was hypothesized, the combination of the *R. horribilis* poison secretion or their fractions plus 25 µM Eto prevented the apoptosis induced by the treatment of only Eto (Figure 8), suggesting that anti-proliferative effect of the *R. horribilis* poison secretion is produced by cell cycle blocking before of G2/M phase.

### 2.6. Effect of Fractions B and C of the R. horribilis PGS on the Migration of Lung Cancer Cells

**Figure 9:**
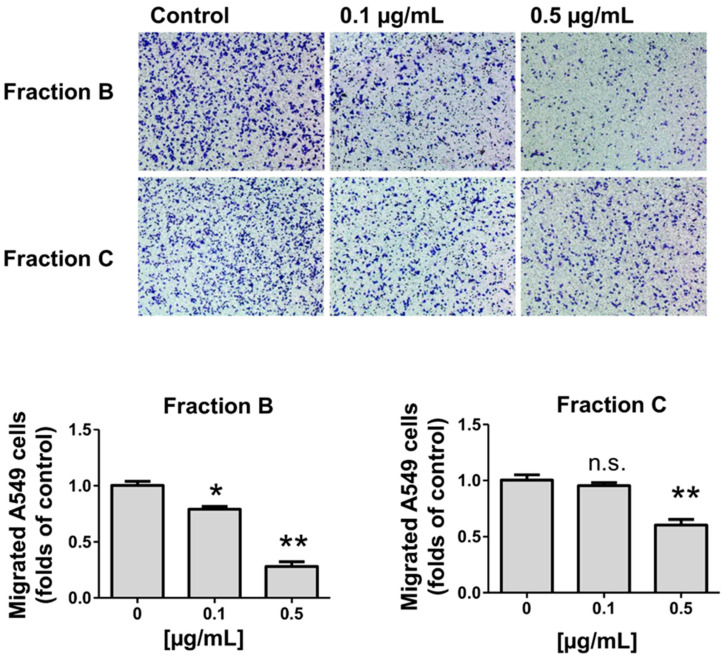
Effect of fractions B and C on fibronectin-dependent migration in A549 cells treated for 24 h. Data shown are the mean ± SEM of two independent experiments. * *p* < 0.05, ** *p* < 0.01, vs. control (DMSO). n.s. not significant.

To evaluate the effect of components of *R. horribilis* poison secretion on the migration of cancer cells, we selected both the most active (fraction B) and the less active (fraction C) on inhibition of proliferation. The lung cancer cells were exposed to both fractions by 24 h and the effect on fibronectin-dependent migration was measured. At 0.1 and 0.5 µg/mL, fraction B reduced the migrated cells to 0.79 ± 0.03 and 0.28 ± 0.04 folds of Control and fraction C decreased the migration to 0.95 ± 0.28 and 0.60 ± 0.05 folds of Control, respectively. As Figure 9 shows, fraction B was more active inhibiting the migration of A549 cells.

Although certain bufadienolides present in the poison secretion of toads produce cell cycle arrest in S-phase [48], reduce the activity and protein levels of topoisomerases [49,50] and intercalates with DNA [51]; other DNA damage-independent mechanisms have also been recently described [8,26]. For example, the major constituents of the PGS of *Rhinella* species such as bufalin, marinobufagin, telocinobufagin, and hellebrigenin exhibit strong binding affinities to tubulin in vitro and in vivo conditions, producing dysregulated microtubule arrangement and G2/M cell cycle arrest in cancer cells [8]. In this work, we identified these same tubulin-inhibiting bufadienolides from *R. horribilis* poison secretions and their fractions, producing irreversible inhibition of proliferation and clonogenic capacity, increasing the ROS production, and inhibiting the fibronectin-dependent migration of human lung cancer A549 cells. An interestingly mechanistic explanation of these effects may be proposed, taking into consideration the effects of current MTA on cancer cells mentioned above [16].

## 3. Conclusions

The composition and biological effects of the PGS of the Amazonian *R. marina* has been extensively studied [8,18,26], while that of *R. horribilis* remained unknown. In this study, some 55 constituents, including argininyl diacids, bufadienolides argininyl diacids, bufadienolides, and alkaloids were identified/tentatively identified in the PGS of *R. horribilis* for the first time. The number of constituents of this species historically confused with *R. marina* [24], was higher than the 29 compounds reported from three Peruvian Amazon populations of *R. marina* [18].

The alkaloids from the venoms are mainly tryptophan derivatives and some of them present anti-inflammatory effects, as shown by Zhang, et al. [52] on Chinese samples. Several studies on the composition of PGS have been undertaken with the methanol soluble fraction of the crude venom. Recently, the effect was reported of PGS of *Bufo bufo* and *R. marina* on metastatic melanoma, finding that the bufadienolide bufalin was the best antiproliferative agent in the samples [53]. Sinhorin, et al. [54] described the composition of the Amazonian *Rhinella margaritifera* PGS and reported argininyl diacids, bufotalin, and cinobufagin argininyl diacids as well as the bufadienolides bufotalin, cinobufagin, and telocinobufagin as main compounds.

The composition of the PGS in the Peruvian species *R. horribilis* differs from that of *R. margaritifera* by the higher ratio of argininyl diesters, a more complex array of oxidized bufadienolides argininyl diesters, the absence of cinobufagin, and the presence of resibufogenin. When compared with the *Rhinella* species from the Paraguayan river basin area, the toad from the northern Peruvian coast has a much lower content of marinobufagin and its argininyl diacids, that are the main compounds in *R. schneideri* and *R. ornata*, while cinobufagin argininyl diesters are relevant for *R. scitula* [9]. Marinobufagin and its argininyl diesters are the main compounds in *R. marina* from the Peruvian Amazon basin [18]. The chemical composition of the Peruvian coast species shows a different profile that may be associated with a different diet and genetic factors. An update on the chemistry, bioactivity, and quality control of the Asian toad *Bufo gargarizans* has been recently published [55] and provides relevant information to be considered for further studies on the South American species.

Although we describe the anti-cancer effect of the *R. horribilis* PGS, some issues remain to be explored, such as the action of the isolated constituents of crude secretion and the effect on non-tumoral cells and in vivo studies. The *R. horribilis* poison secretions produced irreversible inhibition of proliferation and clonogenic capacity, increasing the ROS production, and inhibiting the fibronectin-dependent migration of human lung cancer A549 cells. The fraction B was the most active on proliferative and migration of cancer cells, suggesting that certain constituents present only in this fraction as minor compounds or the combination of the many different compounds acting in synergy may be attractive candidates for further anti-cancer studies.

## 4. Materials and Methods

### 4.1. Parotid Glands Secretions (PGS) Samples

Twenty female *Rhinella horribilis* (Wiegmann, 1833) toads were collected in the Region of Piura, on the north coast of Peru, between December 2018 to January 2019 with herpetologists from Universidad Nacional de Trujillo. The size and weight of the animals ranged between 15.2 to 19.8 cm and 145 to 310 g, respectively. The animals were gently placed in cardboard boxes and then transferred to the Pharmacognosy Laboratory of Universidad Nacional de Trujillo, Peru. The skin secretions were obtained by mild electric stimulation using platinum electrodes (4-ms pulse width, 50 Hz, 5V) (Memex) by ten seconds, as described by Thirupathi, et al. [56]. Next, the parotid glands of the toads were gently pressed with the hands to remove the PGS, which was placed in a beaker. All animals were released after collecting the samples. The fresh PGS (5.4 g) was resuspended in MeOH and was sonicated for 5 min, filtered, taken to dryness under reduced pressure, and lyophilized, affording 900 mg of the crude extract of PGS. The *w*/*w* extraction yield of the MeOH extract was 16.7%.

The obtention of peptides from the toad venom needs a different protocol than that used in our work. The venoms should be extracted with a saline buffer [22] or with acidified water and not with organic solvents. The clean-up requires methods used for protein isolation and identification. The identification of the peptides also requires different analytical tools, MALDI-TOF detection, microsequencing, and synthesis [23].

### 4.2. Thin-Layer Chromatography Analysis

For the thin-layer chromatography (TLC) analysis, commercial plates coated with silica gel F_254_ as the stationary phase were used. For visualization of the TLC profile, the plates were first observed immediately after the TLC at wavelengths (λ) 254 and 365 nm. Afterward, the plates were sprayed with a *p*-anisaldehyde solution reagent freshly prepared (2.0 mL *p*-anisaldehyde in 340 mL ethanol, 40 mL glacial acetic acid, and 20 mL 97% sulfuric acid) followed by heating up to 105 °C for maximum visualization of spots.

### 4.3. HPLC-DAD Analysis

The system used was a Shimadzu^®^ Prominence (Shimadzu Corporation, Kyoto, Japan) consisting of a LC-20AT pump, a SPD-M20A UV diode array detector, CTO-20 AC column oven, and a LabSolutions software (Version 5.51, Shimadzu Corporation, Kyoto, Japan). A MultoHigh 100 RP 18, 5 μ column (250 × 4.6 mm) (CS—Chromatographie Service GmbH, Langerwehe, Germany) was used. The temperature was set at 30 °C. The HPLC analyses were performed using a linear gradient solvent system consisting of methanol:acetonitrile 50:50 (A) and 0.1% formic acid in water (B) with a flow rate of 0.4 mL/min as follows: initial conditions were 8% A and 92% B; 0–10 min: 92% to 85% B; 10–25 min: 85% to 70% B; 25–35 min: 70% to 65% B; 35–45 min: 65% to 55% B; 45–55 min: 55% to 50% B; 55 to 75 min: 50% to 45% B; 75 to 95 min: 45% to 35% B; 95 to 100 min: 35% to 25% B; 100 to 105 min: 25% B; 105 to 110 min: 25% to 40% B; 110 to 120 min: 40% to 60% B; 120 to 130 min: 60% to 75% B; 130 to 135 min: 75% to 92% B; 135 to 145 min: back to 92% B (initial condition). The volume injected was 20 μL. The compounds were monitored at 254, 280, and 330 nm. UV spectra of the compounds detected were recorded from 200 to 500 nm for peak characterization.

### 4.4. HPC-DAD-Q-TOF-MS/MS Analysis

HPLC-DAD-MS/MS analysis was performed using a UHPLC/HPLC-DAD Bruker Elute LC system coupled in tandem with a Q-TOF Compact (Bruker Daltonik, Bremen, Germany). The same above-mentioned reverse phase column and a linear gradient solvent system (HPLC-DAD analysis, Section 4.3) were performed. The volume injected was 5 μL of a 10 mg/mL solution.

The data were acquired in the full scan mode (range of *m*/*z* 50–1500) in positive electrospray ionization mode using capillary voltage at 4.5 kV; nebulizer and drying gas (N_2_) at 4.0 bar and 9.0 L/min, respectively; dry temperature of 200°C. The MS/MS spectra were acquired in auto mode using variable collision energy of 250 to 100% of 10 eV. Internal calibration of the instrument was accomplished using 10 mM sodium formate solution introduced to the ion source via a 20 μL loop at the beginning of each analysis. The compounds were identified by comparison of the fragmentation pattern, UV profile, and mass spectra.

### 4.5. NMR Analysis

The NMR spectra were recorded on a Bruker Avance 400 spectrometer (Bruker, Rheinstetten, Germany) at 400 MHz for ^1^H and 100 MHz for ^13^C in CD_3_OD. Chemical shifts are given in ppm with residual methanol as the internal standard.

### 4.6. Cell Culture Conditions

Human lung cancer A549 cells were grown in Dulbecco’s modified Eagle’s medium (DMEM), containing 25 mM glucose and 4 mM glutamine supplemented with 10% fetal bovine serum (FBS), penicillin (100 IU/mL), and streptomycin (100 µg/mL). No exogenous pyruvate supplementation was added, and cells were maintained in a humidified atmosphere at 37 °C and 5% CO_2_.

### 4.7. Colony Formation

For the colony assay, A549 cells were seeded in 6-well plates at 500 cells per well according to Franken et al. [41] and incubated for 24 h. The cells were treated with the crude extract (L) and fractions A, B and, C of *R. horribilis* secretions for 24 h. After treatment, the medium was replaced with fresh medium, and the cells were incubated for 7 days to allow colony formation. Colonies were stained with 0.5% crystal violet solution in 20% methanol and washed with tap water. Colony formation was analyzed with ImageJ software (NIH, Bethesda, MD, USA) as we described [57].

### 4.8. Determination of ROS Levels

The generation of intracellular oxidative stress was determined using the dihydroethidium (DHE) probe as previously described [58]. In brief, A549 cells were grown in complete media, seeded in 12-well plates (50.000 cells/mL) allowed overnight to attach. Cells were exposed for 24 h to DMSO (Control) or 0.1 and 0.5 µg/mL of crude poison secretion (L) or A, B, and C fractions. Then, culture media was replaced by a solution containing 5 µM DHE in Hank’s balanced salt solution (HBSS) and incubated for 20 min in the dark. After this time, cells were washed, trypsinized, and resuspended in 200 µL of HBSS and measured by FACSCanto flow cytometer.

### 4.9. Migration Assay

Cell migration was evaluated in Boyden Chamber assays (Transwell Costar, 6.5-mm diameter, 8-µm pore size) as previously reported [58]. The cancer cells were exposed to fractions B and C (0.1 and 0.5 µg/mL) for 24 h. Then, cancer cells (50,000 cells/mL) were re-suspended in serum-free medium, plated on top of the chamber insert coated with fibronectin (2 µg/mL), and incubated at 37 °C for 2 h. The inserts were removed, washed, and the bottom side of the inserts was stained with 0.5% crystal violet solution in 20% methanol. Cells from eight different frames were counted for each condition in an inverted microscope.

### 4.10. Annexin V/Propidium Iodide Staining

The cell death was evaluated using annexin V/propidium iodide (AV/PI) dual staining, following the instructions of the Annexin V-FITC Apoptosis Detection Kit (Abcam, Cambridge, UK), as previously reported [57]. In brief, A549 cancer cells were seeded (50.000 cells/mL) in 12-well plates, treated with 0.5 µg/mL of the crude extract (L) and fractions for 24 h. The fluorescence was measured by flow cytometry.

### 4.11. Statistics Analysis

All statistical analyses were performed using Graph Pad Prism 4.03 (GraphPad Software, San Diego, California, USA). The data are expressed as mean ± SEM of three independent experiments. Statistical analysis was performed using one-way or two-way ANOVA with Bonferroni’s post-test for pairwise comparisons. The data were considered statistically significant when *p* < 0.05.

## Figures and Tables

**Table 1:** HPLC-MS/MS data (positive ion mode) of the defense compounds of the lyophilized crude extract of parotoid gland secretion (L) from the Peruvian *Rhinella horribilis* and the fractions 27–30 (A), 31–38 (B), and 39–57 (C). Abbreviations: N° = compound number, for the structures see Figures 3–5; Rt = Retention time; Sample: fractions 27–30 (A), 31–38 (B), 39–57 (C), lyophilized crude extract (L).

N°	Rt (min)	Sample	[M+H]^+^ Detected	[M+H]^+^ Expected	Error (ppm)	MS/MS Fragmentation	Tentative Identification
**1**	11.4–11.9	L	303.1624	303.1663	12.8	303.1624 (<1), 244.1169 (32), 222.1210 (14), 175.1184 (27), 158.0852 (22), 130.1935 (14), 128.0688 (26), 116.0685 (100), 95.0600 (39).	Adipoyl arginine C_12_H_23_N_4_O_5_^+^ [28]
**A1**	12.8	L	176.0685	176.0666	10.8	176.0688 (<1), 159.0660 (17), 143.0720 (9), 133.0536 (16), 117.0560 (52), 115.0533 (100), 115.0533 (100), 105.0673 (31), 89.0368 (17)	Guanidinosuccinic acid C_5_H_10_N_3_O_4_^+^ [28]
**2**	15.6–17.0	A, B, C, L	317.1804	317.1819	4.7	317.1804 (<1), 273.4732 (40), 194.1169 (52), 158.0895 (65), 125.0623 (87), 112.0849 (100)	Pimeloyl arginine C_13_H_25_N_4_O_5_^+^ [28]
**A2**	15.7–17.2	A, B, C, L	203.1178	203.1179	0.5	203.1178 (<1), 188.0932 (50), 173.0708 (100), 145.0764 (9), 118.0667 (10), 89.0364 (16)	Dehydrobufotenine C_12_H_15_N_2_O^+^ [28]
**3**	21.8–22.7	A, B, C, L	331.1962	331.1976	4.2	331.1980 (20), 272.1486 (100), 172.1057 (86), 125.0603 (48), 112.0879 (46)	Suberoyl arginine C_14_H_27_N_4_O_5_^+^ [28]
**5**	25.2–25.5	A, B, C, L	359.2260	359.2289	8.0	359.2366 (21), 272.1458 (100), 246.4343 (17), 226.1422 (20), 203.1506 (33), 116.0996 (35)	Sebacyl arginine isomer C_16_H_31_N_4_O_5_^+^ [28]
**4**	30.9–31.5	A, B, C, L	345.2123	345.2132	2.6	345.2100 (19), 286.1628 (27), 250.1529 (85), 175.1197 (58), 158.0932 (44), 139.0761 (42), 116.0709 (100)	Azelayl arginine C_15_H_29_N_4_O_5_^+^ [28]
**6**	34.0–34.1	A, L	373.2417	373.2445	8.3	373.2341 (34), 286.1592 (100), 254.1458 (30), 203.1485 (40), 172.1096 (40), 116.0702 (75)	Undecadienoyl arginine C_17_H_33_N_4_O_5_^+^ [28]
**24**	48.8–49.3	A, L	745.4000	745.4018	2.4	745.3983 (78), 727.3875 (13), 434.7749 (6), 331.1942 (100), 313.1949 (11), 278.1473 (33), 250.1558 (25), 195.1161 (17), 158.0890 (17), 117.0713 (31), 112.0840 (58), 91.0538 (27)	3-(N-suberoyl argininyl) Hydroxyhellebrigenin isomer 1 C_38_H_57_N_4_O_11_^+^ [28,29]
**19**	49.1–49.2	L	731.4225	731.4226	0.1	331.1932 (100), 314.1670 (18), 278.1441 (25), 175.1128 (10)	3-(N-suberoyl argininyl) Hellebrigenol isomer 1 C_38_H_59_N_4_O_10_^+^ [28]
**14**	49.3–49.7	A, L	715.3888	715.3913	3.5	715.3906 (40), 697.3787 (5), 363.1930 (5), 345.1796 (2), 317.1788 (100), 264.1340 (7), 175.1181 (4), 112.0878 (11)	3-(N-pimeloyl argininyl) Desacetylcinobufotalin C_37_H_55_N_4_O_10_^+^ [32]
**10**	49.8–50.1	A, L	687.3952	687.3964	1.7	687.3984 (46), 669.3618 (4), 385.2361 (5), 303.1627 (100), 268.1292 (11), 250.1171 (13), 105.0685 (12)	3-(N-adipoyl argininyl) Gamabufotalin C_36_H_55_N_4_O_9_^+^ [28,32]
**XIII**	50.6–50.9	A, B, C, L	417.2262	417.2272	2.4	417.2286 (100), 399.2198 (18), 381.2053 (50), 363.1946 (34), 345.1840 (33), 209.0929 (62), 165.0687 (62), 103.0526 (89), 91.0544 (94)	Arenobufagin C_24_H_33_O_6_^+^ [31].
**23**	50.8–50.9	L	731.3845	731.3862	2.3	731.3845 (35), 439.2103 (11), 317.1809 (100), 300.1562 (16), 264.1334 (20), 178.0778 (9), 155.0880 (13)	3-(N-pimeloyl argininyl) Hydroxyhellebrigenin C_37_H_55_N_4_O_11_^+^ [28]
**20**	51.5–51.7	A, B, L	701.3741	701.3756	2.1	701.3747 (96), 683.3639 (11), 665.3641 (2), 381.2004 (3), 364.1916 (5), 363.1984 (4), 335.1984 (18), 317.1910 (3), 303.1640 (100), 268.1239 (14), 250.1194 (17), 175.1192 (3), 129.0689 (16), 91.0528 (31)	3-(N-adipoyl argininyl) Hellebrigenin C_36_H_53_N_4_O_10_^+^ [32]
**36**	52.1–52.4	A, B, L	743.3854	743.3862	2.3	743.3860 (100), 725.3687 (7), 377.1703 (9), 331.1968 (99), 278.1481 (26), 250.1546 (23), 175.1230 (7), 165.0706 (14), 128.0629 (22), 112.0881 (24), 910533 (25)	3-(N-suberoyl argininyl) Hydroxybufotalinin isomer 1 C_38_H_55_N_4_O_11_^+^ [28]
**11**	53.3–54.1	A, B, L	701.4062	701.4120	8.2	701.4080 (95), 683.3979 (2), 385.2287 (4), 317.1767 (100), 264.1285 (33), 236.1363 (8), 105.0669 (20)	3-(N-pimeloyl argininyl) Gamabufotalin C_37_H_57_N_4_O_9_^+^ [28]
**21**	54.1–55.0	A, L	715.3879	715.3913	4.8	715.3864 (100), 697.3839 (7), 669.3725 (3), 381.2027 (5), 364.1992 (1), 363.1928 (5), 651.3676 (6), 353.2058 (2), 345.1816 (3), 335.2013 (8), 317.1787 (71), 282.1417 (8), 264.1309 (14), 236.1364 (9), 175.1165 (8), 112.0861 (23), 105.0702 (17), 91.0535 (27)	3-(N-pimeloyl argininyl) Hellebrigenin C_37_H_55_N_4_O_10_^+^ [32]
**19**	54.2–54.6	A, L	731.4171	731.4226	7.5	731.4143 (72), 363.1946 (12), 331.1891 (100), 278.1446 (35), 250.1450 (15), 175.1177 (17), 112.0873 (23)	3-(N-suberoyl argininyl) Hellebrigenol isomer 2 C_38_H_59_N_4_O_10_^+^
**13**	54.3–54.8	A, B, L	701.3728	701.3756	4.0	701.3721 (100), 684.3766 (1), 610.9012 (1), 399.2135 (6), 381.2023 (2), 353.2069 (2), 335.1959 (1), 303.1635 (89), 286.1387 (9), 268.1256 (15), 250.1156 (17), 175.1180 (11), 116.0692 (16), 112.0878 (15), 105.0691 (13), 91.0537 (10)	3-(N-adipoyl argininyl) Arenobufagin C_36_H_53_N_4_O_10_^+^ [32]
**36**	54.7–54.9	A, L	743.3825	743.3862	4.9	743.3829 (65), 349.1888 (8), 331.1935 (100), 296.1560 (9), 278.1480 (25), 250.1528 (55), 233.0955 (4), 179.0855 (11), 112.0869 (34), 91.0555 (22)	3-(N-suberoyl argininyl) Hydroxybufotalinin isomer 2 C_38_H_55_N_4_O_11_^+^ [28]
**34**	55.1–55.4	A, B, L	713.3706	713.3756	7.0	713.3715 (100), 695.3598 (3), 667.3739 (4), 317.1781 (88), 300.1509(22), 282.1434(12), 264.1334 (12), 236.1370 (16), 209.0860 (5), 179.0818 (10), 175.1225 (7), 112.0855 (35), 91.0524 (43)	3-(N-pimeloyl argininyl) Bufotalinin C_37_H_53_N_4_O_10_^+^ [28]
**VI**	56.5–56.7	A, B, L	419.2400	419.2428	6.6	419.2400 (5), 401.2318 (30), 365.2088 (63), 347.2036 (36), 319.1615 (33), 213.1616 (27), 91.0531 (51)	Hellebrigenol C_24_H_35_O_6_^+^ [32]
**IV or V**	57.0–57.6	A, B, C, L	417.2244	417.2272	6.7	417.2226 (100), 399.2122 (35), 371.2176 (4), 353.2056 (3), 335.1972 (5), 255.0976 (2), 175.0722 (4)	Bufarenogin or ψ-Bufarenogin C_24_H_33_O_6_^+^ [32]
**19**	57.0–57.8	A, L	731.4184	731.4226	5.7	731.4189 (55), 713.4048 (5), 683.3909 (10), 665.3843 (4), 331.1919 (100), 278.1468 (10), 250.1475 (9), 175.1179 (4), 112.0854 (20)	3-(N-suberoyl argininyl) Hellebrigenol isomer 3 C_38_H_59_N_4_O_10_^+^ [28]
**16**	57.9–58.2	A, L	687.3947	687.3964	2.4	687.3960 (27), 669.3813 (7), 303.1641 (100), 250.1177 (13), 175.1446 (8), 145.0968 (14), 91.0533 (17)	3-(N-adipoyl argininyl) Telocinobufagin C_36_H_55_N_4_O_9_^+^ [28]
**24**	58.1–58.4	A, L	745.4001	745.4018	2.3	745.4017 (11), 683.4230 (11), 345.2155 (10), 331.1923 (100), 303.1952 (8), 278.1492 (10), 175.1148 (15), 112.0836 (35)	3-(N-suberoyl argininyl) Hydroxyhellebrigenin isomer 2 C_38_H_57_N_4_O_11_^+^ [28]
**30**	58.3–59.0	A, B, C, L	685.3780	685.3807	3.9	685.3757 (28), 669.3790 (5), 667.3653 (3), 365.2086 (3), 349.2058 (4), 303.1615 (100), 268.1240 (12), 250.1140 (12), 175.1140 (6), 105.0678 (13)	3-(N-adipoyl argininyl) Marinobufagin C_36_H_53_N_4_O_9_^+^ [28]
**22**	58.4–60.0	A, L	729.4021	729.4069	6.5	729.4010 (100), 711.3899 (8), 693.3760 (2), 683.3970 (2), 363.1912 (4), 331.1926 (68), 278.1459 (16), 250.1516 (16), 175.1188 (8), 112.0861 (27), 91.0534 (27)	3-(N-suberoyl argininyl) Hellebrigenin C_38_H_57_N_4_O_10_^+^ [28,29]
**XIV**	59.0–59.6	A, B, C, L	417.2242	417.2272	7.2	417.2244 (62), 399.2122 (26), 363.1903 (30), 353.2077 (26), 335.1959 (73), 317.1874 (10), 275.1754 (16), 211.1433 (33), 128.0608 (71), 91.0527 (100)	Hellebrigenin C_24_H_33_O_6_^+^ [28]
**31**	60.5–64.3	A, L	699.3980	699.3964	2.3	699.3971 (38), 681.3823 (7), 365.2102 (4), 347.2001 (4), 317.1806 (100), 264.1314 (11), 236.1361 (6), 175.1198 (5), 112.0883 (10), 91.0523 (10)	3-(N-pimeloyl argininyl) Marinobufagin C_37_H_55_N_4_O_9_^+^ [28]
**XII**	60.8–61.4	A, B, C, L	415.2092	415.2115	5.5	415.2085 (22), 379.1865 (8), 361.1792 (12), 351.1927 (30), 333.1820 (26), 237.1619 (28), 165.0669 (30), 128.0609 (64), 115.0530 (79), 91.0534 (100)	Bufotalinin C_24_H_31_O_6_^+^ [32]
**17**	62.4–64.7	A, B, L	701.4097	701.4120	3.3	701.4087 (24), 683.3955 (5), 349.2203 (3), 317.1786 (100), 264.1308 (5), 236.1406 (3), 175.1172 (3), 105.0711 (8)	3-(N-pimeloyl argininyl) Telocinobufagin C_37_H_57_N_4_O_9_^+^ [28]
**33**	62.7–63.7	A, B, C, L	683.3682	683.3651	3.6	683.3613 (100), 665.3521 (2), 335.1963 (4), 303.1635 (40), 286.1371 (9), 268.1273 (8), 250.1161 (17), 226.1057 (3), 175.1187 (5), 112.0873 (13)	3-(N-adipoyl argininyl) Marinobufagin-9,11-ene C_36_H_51_N_4_O_9_^+^
**II**	62.9–63.4	A, B, C, L	403.2453	403.2479	6.4	403.2445 (30), 385.2335 (58), 349.2120 (44), 337.2111 (41), 275.1742 (25), 253.1888 (39), 241.1922 (49), 161.0968 (29), 105.0785 (46), 91.0546 (100)	Gamabufotalin C_24_H_35_O_5_^+^ [28,32]
**VIII**	64.0-64.7	A, B, C, L	401.2312	401.2323	2.7	401.2295 (34), 383.2866 (3), 365.2081 (15), 347.1970 (20), 337.2165 (14), 319.2024 (10), 257.1163 (21), 239.1059 (32), 211.1096 (27), 183.1154 (24), 128.0626 (86), 105.0700 (85), 91.0547 (100)	Resibufaginol C_24_H_33_O_5_^+^ [31]
**15**	64.4–66.5	A, B, C, L	729.4052	729.4069	2.3	729.4077 (100), 399.2156 (5), 381.2065 (2), 363.1959 (2), 353.2100 (1), 345.1862 (1), 335.1983 (2), 331.1959 (83), 278.1487 (25), 250.1541 (18), 175.1200 (11), 112.0875 (27).	3-(N-suberoyl argininyl) Desacetylcinobufotalin C_38_H_57_N_4_O_10_^+^ [29]
**12**	66.4–67.2	A, B, C, L	715.4254	715.4277	3.2	715.4250 (80), 697.4150 (12), 349.2108 (4), 331.1936 (100), 314.1679 (12), 278.1462 (32), 250.1514 (24), 175.1165 (9), 116.0691 (22), 91.0531 (16)	3-(N-suberoyl argininyl) Gamabufotalin C_38_H_59_N_4_O_9_^+^ [28]
**XI**	66.5–66.7	B, L	415.2090	415.2115	6.0	415.2054 (100), 397.1832 (3), 333.1887 (3), 283.1621 (5), 253.0760 (5), 175.0999 (6)	19-Oxo-desacetylcinobufagin C_24_H_31_O_6_^+^ [28]
**28**	66.5–69.5	A, B, C, L	697.3766	697.3807	5.9	697.3745 (100), 679.3595 (2), 363.1933 (3), 335.1966 (4), 317.1759 (61), 264.1290 (33), 236.1349 (11), 175.1154 (4),	3-(N-pimeloyl argininyl) Resibufagin C_37_H_53_N_4_O_9_^+^ [28]
**18**	68.6–72.0	A, B, C, L	715.4265	715.4277	1.7	715.4266 (44), 697.4156 (7), 349.2136 (4), 331.1944 (100), 314.1708 (3), 278.1484 (8), 250.1538 (5), 175.1193 (4), 105.0695 (11), 91.0540 (11)	3-(N-suberoyl argininyl) Telocinobufagin C_38_H_59_N_4_O_9_^+^ [28]
**32**	70.0–72.6	A, B, C, L	713.4101	713.4120	2.6	713.4100 (48), 695.3992 (5), 365.2075 (3), 331.1941 (100), 314.1681 (5), 296.1581 (3), 278.1473 (12), 250.1533 (8), 175.1198 (8), 112.0873 (16), 91.0536 (15)	3-(N-suberoyl argininyl) Marinobufagin C_38_H_57_N_4_O_9_^+^ [28]
**X**	71.8–72.6	A, B, C, L	399.2150	399.2166	4.0	399.2150 (<1), 381.2104 (24), 363.2394 (14), 331.1949 (35), 275.1786 (19), 239.1741 (52), 213.1297 (33), 128.0661 (46), 115.0554 (42), 105.0684 (67), 91.0533 (100)	Resibufagin C_24_H_31_O_5_^+^ [32]
**29**	74.6–78.9	A, B, C, L	711.3941	711.3964	1.1	711.3956 (100), 363.1933 (3), 331.1937 (40), 278.1463 (24), 250.1522 (18), 175.1179 (14)	3-(N-suberoyl argininyl) Resibufagin C_38_H_55_N_4_O_9_^+^ [28]
**7**	74.6–75.8	A, B, L	671.4014	671.4014	0.0	671.4048 (64), 653.3916 (8), 351.2269 (5), 303.1638 (100), 286.1403 (6), 250.1151 (18), 175.1213 (3), 158.0926 (4), 105.0691 (13), 91.0525 (14)	3-(N-adipoyl argininyl) bufalin C_36_H_55_N_4_O_8_^+^ [28]
**III**	75.1–76.0	A, B, C, L	403.2464	403.2479	3.7	403.2456 (57), 385.2353 (37), 367.2252 (44), 349.2135 (79), 253.1929 (31), 215.1780 (30), 105.0700 (79), 91.0537 (100)	Telocinobufagin C_24_H_35_O_5_^+^ [29,31]
**35**	76.4–77.5	A, B, L	727.3922	727.3913	4.0	727.3917 (100), 711.3898 (6), 397.1995 (9), 331.1962 (100), 278.1494 (35), 250.1532 (29), 175.1180 (21), 112.0885 (34), 91.0540 (23)	3-(N-suberoyl argininyl) Bufotalinin C_38_H_55_N_4_O_10_^+^ [28]
**IX**	78.3–79.2	A, B, L	401.2323	401.2323	0.0	401.2335 (23), 383.2235 (10), 365.2105 (51), 347.2012 (32), 337.2162 (17), 319.2068 (19), 269.1890 (20), 253.1938 (44), 251.1797 (31), 239.1061 (22), 183.1157 (25), 128.0639 (74), 105.0710 (81), 91.0543 (100)	Marinobufagin C_24_H_33_O_5_^+^ [32]
**25**	82.6–86.3	A, B, C, L	669.3840	669.3858	2.7	669.3835 (100), 349.2124 (2), 303.1628 (79), 286.1395 (5), 268.1264 (14), 250.1156 (24), 175.1188 (8), 116.0695 (11), 91.0531 (6)	3-(N-adipoyl argininyl) Resibufogenin C_36_H_53_N_4_O_8_^+^ [28]
**8**	83.0–86.2	A, L	685.4142	685.4171	4.2	685.4113 (94), 667.4030 (11), 351.2260 (7), 317.1779 (100), 300.1539 (10), 264.1298 (24), 175.1181 (9), 112.0860 (22), 91.0520 (20)	3-(N-pimeloyl argininyl) bufalin C_37_H_57_N_4_O_8_^+^ [28]
**I**	87.8–88.7	A, B, C, L	387.2512	387.2530	4.6	387.2482 (60), 369.2396 (72), 351.2291 (52), 333.2204 (10), 255.2101 (57), 187.1454 (14), 173.1330 (16), 128.0637 (36), 105.0698 (88). 91.0541 (100)	Bufalin C_24_H_35_O_4_^+^ [31]
**26**	91.4–94.6	A, L	683.4000	683.4014	2.0	683.4015 (100), 665.3974 (2), 349.2114 (5), 317.1791 (83), 300.1531 (11), 282.1435 (11), 264.1338 (25), 236.1358 (13), 175.1163 (10), 143.0834 (6), 112.0868 (16), 91.0546 (12).	3-(N-pimeloyl argininyl) Resibufogenin C_37_H_55_N_4_O_8_^+^ [28]
**9**	91.4–95.1	A, B, C, L	699.4313	699.4327	2.0	699.4311 (100), 681.4213 (14), 351.2279 (6), 331.1942 (78), 314.16814 (9), 296.1568 (6), 278.1477 (21), 250.1522 (14), 175.1169 (9), 112.0863 (21), 91.0541 (15)	3-(N-suberoyl argininyl) bufalin C_38_H_59_N_4_O_8_^+^ [28]
**VII**	97.3–98.3	A, B, C, L	385.2328	385.2373	7.7	385.2318 (8), 367.2221 (15), 349.2133 (12), 321.2151 (6), 253.1923 (19), 241.1195 (14), 205.0860 (16), 185.0928 (4), 115.0544 (49), 105.0689 (69), 91.0531 (100)	Resibufogenin C_24_H_33_O_4_^+^ [29,32]
**27**	98.7–103.1	A, B, C, L	697.4117	697.4171	7.7	697.4110 (100), 679.4003 (2), 349.2108 (3), 331.1917 (85), 314.1667 (7), 278.1455 (26), 250.1503 (19), 175.1157 (9), 112.0854 (19), 910525 (11)	3-(N-suberoyl argininyl) Resibufogenin C_38_H_57_N_4_O_8_^+^ [28]

**Table 2:** Anti-proliferative effect of fractions A, B, and C and crude extract of parotoid gland secretion of *R. horribilis* on lung cancer A549 cells. Data are expressed as IC_50_ values (µg/mL ± SEM) of three independent experiments.

Time	Crude Extract	Fraction A	Fraction B	Fraction C
24 h	0.031 ± 0.007	0.010 ± 0.001	0.007 ± 0.001	0.025 ± 0.001
48 h	0.015 ± 0.001 ^a^	0.011 ± 0.003	0.006 ± 0.001	0.007 ± 0.002 ^a^

^a^ Significant difference compared to the same fraction (*t*-test two-tailed, IC 95%).
